# The limits of Humeanism

**DOI:** 10.1007/s13194-018-0205-9

**Published:** 2018-05-16

**Authors:** Jesse M. Mulder

**Affiliations:** Utrecht, The Netherlands

**Keywords:** Humeanism, Laws of nature, Metaphysics, Modality, Material objects

## Abstract

Humeans take reality to be devoid of ‘necessary connections’: things just happen. Laws of nature are to be understood in terms of what ‘just happens’, not vice versa. Here the Humean needs some conception of what it is that ‘just happens’ – a conception of the *Humean mosaic*. Lewis’s Humeanism incorporates such a conception in the form of a Lewis-style metaphysics of objects, properties, and modality. Newer versions of Humeanism about laws of nature, such as the Better Best Systems approach (BBS), typically reject such a Lewisian metaphysics, but it remains unclear what they can offer in its place. By exploring different candidate conceptions, this paper sheds light on the limits of Humeanism about laws of nature: not *all* conceptions of the Humean mosaic form a suitable basis for a Humean theory of laws. In fact, only a metaphysics roughly in line with Lewis’s will do. The paper ends with a tentative generalization of this result, thus pointing to the ‘limit’ of Humeanism in general: taking the Humean way of thinking to its limit results in a rejection of the whole idea of such a mosaic – and hence of Humean mosaic-based accounts of anything.

A Humean attitude towards topics such as causality and laws of nature starts with strong suspicions about what Hume called “necessary connections”: “there are no ideas, which occur in metaphysics, more obscure and uncertain” (Hume 2007, VII.1, p. 45). It ends with analyses of the relevant notions that do not rely on such necessary connections. In reality, it is thought, things just happen, there is no ‘causal glue’ that ties different events together, no laws that ‘govern’ the behavior of objects.^1^ Rather, causality and laws are to be accounted for in terms of the things that just happen, not vice versa.

But what does it mean to say that ‘things just happen’? Arguably, one could read Hume here epistemologically, i.e., such that what *the senses* provide us with ‘just happens’, that they reveal to us no ‘necessary connections’ – all we have to go on is a mere succession of impressions.^2^ However, here I want to focus on what is known under the label of contemporary Humean (or neo-Humean) theories of, e.g., laws of nature and causality, and these understand themselves not primarily as epistemological doctrines in this way, but rather as theories that build on regularities among what objectively happens in the ‘mind-independent world’.^3^

The most explicit formulation of this sort of ‘metaphysical’ Humeanism can be found in David Lewis, who dubbed it ‘Humean Supervenience’:

Humean supervenience … is the doctrine that all there is to the world is a vast mosaic^4^ of local matters of particular fact, just one little thing and then another. … We have geometry: a system of external relations of spatiotemporal distance between points. Maybe points of space-time itself, maybe point-sized bits of matter or aether or fields, maybe both. And at those points we have local qualities: perfectly natural intrinsic properties which need nothing bigger than a point at which to be instantiated.^5^ For short: we have an arrangement of qualities. And that is all. There is no difference without difference in the arrangement of qualities. All else supervenes on that. (Lewis 1986b, pp. ix–x)

Lewis indeed analyses causality and laws of nature in terms of the Humean mosaic, as he conceives of it here, i.e., in terms of the “arrangement of qualities”. In particular, for him the laws depend only on the *actual* arrangement of qualities (viz., our world): they provide the best summary of that arrangement. Causality, on Lewis’s counterfactual approach, is then understood to depend on how the qualities are arranged not only in our own world but also in *relevant* other possible worlds, where the relevance is captured using the actual laws (amongst other things).

But not all Humeans agree with Lewis on the nature of the mosaic. This raises the question: which conceptions of the Humean mosaic are suitable for a Humean project? Answering this question may help identify certain limits of the Humean project – only a certain limited range of conceptions of the Humean mosaic will be suitable for the sorts of Humean views about laws of nature or causality that one finds in the contemporary debate. Now, I can’t fully answer this question, and determine the relevant limits of Humeanism, within the context of this article. But I do aim to take a first step. In particular, I will focus in what follows on the possibility of conceptions of the mosaic that differ from Lewis’s as suggested by defenders of the recent so-called ‘Better Best Systems’ (BBS) theory of laws. Still, as I hope to make clear in the afterthoughts at the end of this paper (§4), the results may be taken to point in the direction of a more comprehensive estimation of the limits of Humeanism (in the mentioned sense).

Thus, I start in §1 below by looking more closely at Humeanism about laws of nature, in particular at Lewis’s ‘orthodox’ Best Systems view, with an eye to identifying the elements that are relevant for the underlying conception of the Humean mosaic. In §2 I then focus on the Humean mosaic proper by considering the BBS variant of Humeanism about laws, which explicitly departs from the Lewisian view, and suggests to replace it with an ‘explosive realism’. I consider various conceptions of the Humean mosaic along ‘explosive realist’ lines that might be thought to provide suitable bases for this Humean theory. Having dismissed certain shapes this conception might take in §2, I then turn in §3 to what one might call ‘moderately’ Better Best Systems – the one variant conception of the mosaic that survived §2’s criticisms. This variant stays rather close to Lewis’s views in the relevant respects (hence ‘moderately’). Finally, in §4, I offer the promised afterthoughts concerning the limits of Humeanism on the basis of the considerations so far. In particular, I sketch two such limits: first, that the Humean will have to stay in the vicinity of a Lewis-like conception of the mosaic if his project is to make sense at all; and second, that there is some pressure, when considering the motivation for the Humean enterprise, to abandon the very idea of a Humean mosaic exhibiting regularities, and thus to entirely abandon the orthodox Humean project of accounting for laws (or for anything whatsoever, for that matter) in terms of such regularities.

## Humeanism about laws

The Humean approach to laws of nature attempts to account for laws using materials Hume would find acceptable – viz., a Humean mosaic. A ‘naïve’ Humean takes laws to be just regularities within the mosaic: it is a law that *A*s are *B* just if *A*s are always *B*. This move, as has been frequently observed, reverses the order of explanation: whereas intuitively, the laws are to explain the behavior of things, the Humean has to say that the behavior of things, taken together, explains the laws. (Humeans would typically take this not as a problem for, but rather as a statement of, their view.) This reversal creates the single most important challenge for Humeanism: to distinguish those generalizations that are truly ‘lawlike’ from other, merely accidental generalizations. After all, the non-Humean can simply start with laws and use them to single out the law-like generalizations (assuming she still wants to deal in terms of generalizations at all, which is false for quite some non-Humean views – Cartwright (1984) being a case in point). Accordingly, the history of Humeanism consists in large part in attempts to meet this challenge (even if not in these precise words).^6^

As said, Lewis’s ‘Best Systems’ account appears to be the most promising candidate Humean account of laws of nature at present. It is also known as the Mill-Ramsey-Lewis (MRL) account.^7^ Here is a concise statement of its core thesis:

We can restate Ramsey’s 1928 theory of lawhood as follows: a contingent generalization is a law of nature if and only if it appears as a theorem (or axiom) in each of the true deductive systems that achieves a best combination of simplicity and strength. (Lewis 1973, p. 73)

The proposal is to have all possible summaries of the Humean mosaic compete with each other. Such summaries are assumed to take the shape of deductive systems with certain axioms, and the systems that score best on the balance between simplicity and strength come out as winners: they are the ‘Best Systems’. Simplicity is cashed out, for example, in terms of some complexity metric, whereas strength is understood in terms of the amount of information embodied in the axioms – stronger systems say more about the entire mosaic.^8^

As Lewis observes, it is not even required, on the Best Systems approach, to restrict the laws to *general* truths: if it improves the overall balance of strength and simplicity, a truth about particular aspects of the entire mosaic may be regarded as a law as well.^9^

What Lewis does require, however, is that we run the Best Systems competition on the basis of a predicate-logical language containing a certain privileged set of predicates. Without that restriction, so he rightly thinks, the whole project becomes trivial: we could simply choose a language, say, with a primitive predicate *P* which is stipulated to apply to an object just if it is part of a world which is composed of matters of fact exactly similar to those that make up the actual mosaic. The generalization ‘Everything is *P*’ would then easily win the competition because it is maximally strong and (arguably) maximally simple – but also very uninteresting. Now, Lewis’s way of restricting the language for which we run the Best Systems machinery is by endorsing a metaphysically privileged set of ‘perfectly natural properties’: those are the fundamental properties that are instantiated by the basic building blocks of the universe – “maybe points of space-time itself, maybe point-sized bits of matter or aether or fields, maybe both”, as I quoted Lewis above.^10^

In fact, the privileged set of natural properties is already required to arrive at a non-trivial conception of the Humean mosaic in the first place: an abundant construal of properties, on which *any* set of objects amounts to a property, trivializes the whole idea of *regularities* within a mosaic. On such a construal it will be more appropriate to speak of *drawing* patterns into the mosaic than of *recognizing* patterns in it.^11^

Hence, the (predicate-logical) language on which the Best Systems competition is to be based should include primitive predicate symbols for just the ‘perfectly natural properties’; contrived ones (like our *P*) are not allowed. In this way, the Best Systems theory also avoids the allegedly impossible task of having to compare the balance of simplicity and strength for systems couched in different vocabularies.^12^

Following Lewis, many have found it natural to identify this set of perfectly natural properties with the fundamental properties of (future) physics.^13^ Yet this creates a problem with regard to the laws of the other sciences (assuming that they trade in a vocabulary different than that of physics), unless one is prepared to accept that only the laws of physics are ‘true’ laws of nature. But not everyone is prepared to go that way, and this is one of the motivations for the recently proposed amended version of the Best Systems approach mentioned earlier: the so-called ‘Better Best Systems’ (BBS) approach.^14^ On this approach, each science has its own laws resulting from a Best Systems competition based on the specific vocabulary of the science in question. It is thought that this amendment to the Best Systems approach makes room for autonomous special sciences.^15^

## The underlying Humean mosaic

I have introduced the Best Systems approach, and its recent ‘better’ variant, as a sort of culmination of Humeanism about laws, i.e., as the most promising member of a family of views that does not take the laws to govern what happens, but rather takes them to summarize what happens (either in simple regularities, or in the more complicated Best Systems-fashion that Lewis made popular). This family of views thus builds on the idea of a Humean mosaic: the mosaic is what is given, what needs to be adequately summarized if we want to arrive at laws. Now, what does the mosaic have to look like in order to provide a useful foundation for this type of approach? Most of the defenders of a Humean theory of laws do not develop a detailed conception of this underlying mosaic – except, of course, for Lewis, whose metaphysical views are developed in considerable detail. Let us review his conception of the mosaic, so that we can then see in which ways the BBS approach might diverge from that conception.

As we saw, Lewis’s conception of the mosaic is somewhat open still; it depends on what the bearers of the most fundamental (perfectly natural) properties turn out to be. Perhaps these are simply the space-time points themselves,^16^ perhaps they are indivisible, point-sized bits of matter, maybe fields of some sort. For concreteness, let us assume that the mosaic consists of point-sized bits of matter; and for convenience, let us assume that every space-time point houses exactly one such point-sized ‘bit’. (Except for the ease of exposition in what follows, nothing hangs on this.)

This gives us a basic conception of the mosaic. On Lewis’s view, composite objects then come ‘for free’: they are simply mereological sums of such basic, point-sized bits. Let us call such sums (Lewisian) *material objects*. To each region of space-time there corresponds one and only one such object, and this means, for Lewis, that material objects *perdure* (if their region is extended in time): they persist by having distinct temporal parts at each time at which they exist. Material objects are thus individuated by their spatiotemporal boundaries or, equivalently, by their (proper) parts.^17^ Different region, different object.

For Lewis, this does not mean, however, that such objects *could not have had different spatiotemporal boundaries* or that they *could not have been composed by different parts*: these modal statements are to be analyzed using his counterpart-theoretic framework, according to which it is possible for something to be larger than it actually is (say) just if there is a possible world in which its counterpart is in fact larger. In short, then, a thing’s non-modal qualities are determined by the distribution of perfectly natural properties over the region of space-time it occupies, while a thing’s modal features (including its dispositions etc.) depend on how things stand with its counterparts in relevant possible worlds – where relevance is, again, determined in part by what the laws are.^18^

So, on this Lewisian picture we must distinguish carefully between, on the one hand, the modally ‘thin’ conception of objects as inhabiting his Humean mosaic by being mereological sums of its basic, point-sized material ingredients, which in turn function as the basis for his Humean account of laws, and, on the other hand, a subsequent modally ‘thick’ conception of those objects as, for instance, rabbits, stars, electrons, and water molecules (I will give more content to this idea of modal ‘thickness’ below, and defend it further in §3). This is to say that Lewis’s system embodies a reduction of those modally ‘thick’ objects to the modally ‘thin’ ones: rabbits, for instance, *just are* material objects satisfying certain qualitative and modal conditions. This assumes that the qualitative and modal conditions can be fully separated in the final analysis.^19^ And so *in the end* it doesn’t matter for Lewis whether we say that the mosaic is inhabited by modally thick rabbits and the like, or rather by the modally thin material objects (since the former are reduced to the latter anyway). But it *does* matter at the beginning, so to speak. For, the reduction of (modally ‘thick’) rabbits etc. to (modally ‘thin’) material objects only works (if it works at all) if the mosaic, and the laws, can be specified in ‘rabbit-free’ terms. Thus it transpires that the modally thin conception of the basic elements of the mosaic (point-sized bits of matter) is *fundamental*: it provides the basis for the reduction.^20^ Finally, this reduction crucially *rests on the laws of nature*: these determine which worlds are relevant for settling the modal features of things. – I will return to this intricate structure of Lewis’s Humeanism below, where we will see that this clean separation of the qualitative from the modal has important consequences for the space of Humeanly acceptable conceptions of the mosaic.

Now, as I said, not everyone accepts such a Lewisian construal of the Humean mosaic. Callender and Cohen, for instance, regard it to be a virtue of their Better Best Systems view that it is *not* committed to Lewisian metaphysics across the board. Instead, they propose ‘explosive realism’:

[T]he world permits possibly infinitely many distinct carvings up into kinds, each equally good from the perspective of nature itself, but differentially congenial and significant to us given the kinds of creatures we are, perceptual apparatus we have, and (potentially variable) matters we care about. (Cohen and Callender 2009, p. 22)^21^

The thought seems to be that ‘the world’, that is, the Humean mosaic, simply does not settle the question which predicates we should start with when we want to look for laws. *Pace* Lewis, there is no metaphysically privileged set of allegedly ‘perfectly natural’ properties. By thus allowing to run Best Systems competitions based on many different sets of properties, this proposal makes room for the special sciences to each, autonomously, run its own Best Systems competition without having to worry about how this enterprise relates to the supposed ‘perfectly natural properties’.

However, by focusing solely on the *properties* that are or are not ‘allowed’, this way of thinking leaves out one important issue: the *objects* that occupy the mosaic.^22^ What are the implications of explosive realism for the conception of the objects that inhabit the underlying mosaic?

This question is especially important if one wants to replace Lewis’s conception of the mosaic, in terms of point-sized bits of matter displaying patterns of perfectly natural properties, by a conception in terms of the various vocabularies of the various sciences, as Cohen and Callender seem to propose. For it is not to be expected that these various sciences conceive of the *bearers* of the various properties they are working with as Lewisian material objects. Their conception of these objects may well be what I call modally ‘thick’. I will gradually develop this point, and the complications it gives rise to with respect to the conception of the mosaic, in what follows, and I will attempt a further defense of the idea of modal ‘thickness’ in §3.

So let us start our inquiry into alternative (non-Lewisian) conceptions of the mosaic by taking up Cohen and Callender’s suggestion: explosive realism. We will have to distinguish between different ways in which one might intend to ‘explode’ reality in order to arrive at an ‘explosive realism’. Initially, two such ways suggest themselves. The first, which I will call *substantial explosive realism*, replaces the fundamental conception of the mosaic in terms of a modally thin notion akin to Lewis’s point-sized bits of matter with a fundamental conception of the mosaic in much less austere terms. In fact, no limit whatsoever is placed on the terms in which the objects inhabiting the mosaic are to be fundamentally understood – that’s the ‘explosion’. The second reading of explosive realism, which I will call *deflationary explosive realism*, keeps the fundamental conception of the mosaic in terms of a modally thin notion akin to Lewis’s point-sized bits of matter intact, but stresses that this mosaic ‘permits’ (as Cohen and Callender put it) a wide range of ‘carvings’ into various kinds of objects. So here the explosion takes place not at the fundamental level but rather at the level of our ‘carving operations’.

Consider the first option, which is, to be fair, not likely to coincide with what Cohen and Callender have in mind. The mosaic is, on this proposal, not to be conceived, fundamentally, in terms of Lewis-style point-sized bits of matter only. Rather, the proposal is to ‘explode’ the fundamental conception of the mosaic, so that it includes also modally ‘thick’ things like rabbits, tables, water molecules, mountains, oak trees – and many, many unheard-of things.^23^ – Of course, as I said, Lewis’s mosaic can be said to contain all of these as well. But it bears emphasizing that they do not play a role in Lewis’s *fundamental conception* of the mosaic: there are rabbits in the mosaic because there are suitable *material objects* (in his sense) that display a leporine distribution of qualities and stand in appropriate relations to their counterparts in certain other possible worlds. For Lewis the sortal concept *rabbit* is not fundamental in the way his concept of point-sized bits of matter is: to be a rabbit *just is* to be a sum of such bits of matter (viz., a material object) satisfying certain qualitative and modal conditions. Hence, fundamentally speaking, the Lewisian mosaic is not *composed* of things like rabbits. It rather ‘permits’ them by providing suitable reductive bases. In other words: the Lewisian mosaic has room for rabbits *only if* the sketched reductive analysis can be pulled off; the fundamental conception of the mosaic is to provide the basis for that reduction. Substantial explosive realism is radically different: it is *fundamentally* composed of rabbits and the like; reduction is then out of the question.

So, asserting that the mosaic *itself* is, fundamentally, composed of such modally ‘thick’ things as rabbits etc., thus giving up on a reductive approach to *all* of these kinds, elevates all of them to the status of fundamental sortal concepts – the status *point-sized bit of matter* enjoys in Lewis’s framework. For, as soon as we reductively explain the presence of, say, rabbits in terms of more basic things’ fulfilling certain conditions, as on the Lewisian view, we have thereby removed them from our fundamental conception of the mosaic – we are, then, using a (perhaps tacitly) preformed conception of the mosaic to reductively account for the presence of rabbits.^24^ Thus, taking the mosaic itself to include the full ‘explosion’ results in an incredibly wealthy catalogue of fundamental kinds of things (a maximally wealthy catalogue, in fact). That is not all by itself a problem, of course (unless one is biased toward desert landscapes), but it does create a tension with the very idea of a Humean mosaic, which is supposed to be free of necessary connections. Let us see why.

The concept of a rabbit, I said, is modally ‘thick’. For instance, rabbits don’t simply pop into existence; they come from their parents. A rabbit only stays alive as long as it takes in suitable nourishment, meaning that in its past there has to have been enough such nourishment in its proximity. Rabbits don’t turn into butterflies, meaning that in the future it will not suddenly transform into one. It is clear, then, that if something *here-now* is a rabbit, this has lots of implications for how things are at other places and times: there have to have been parents, nourishment, absence of conditions too extreme for rabbits to survive, etc. In other words, if something here-now in the mosaic is a rabbit, and *rabbit* is a fundamental sortal concept, then the mosaic contains ‘necessary connections’. (Note that this only holds because the modally thick concepts are taken to be fundamental!) Nor does it make a difference to change the example to one drawn from physics, rather than biology: electrons, like rabbits, don’t pop into existence either; they don’t suddenly change from being negatively charged to being positively charged (or to being 20 m in diameter); when shot into (regular) cloud chambers they do not trace out the word ‘electron’, etc. These necessary connections threaten to make the Humean, regularity-based approach to laws of nature superfluous: we can simply list the necessary connections that we already have within the mosaic.^25^ In short, this conception of the mosaic is quite substantial indeed; hence *substantial explosive realism*. (There is an apparent way out here for the Humean; I discuss it in the next section.)

The problem lies with the conceptual connections that I have boldly claimed to be inherent in the concept *rabbit* (or *electron*). By elevating such concepts to fundamental concepts, these conceptual connections are turned into necessary connections – reduction (or elimination) is out of the question. So, one might think, the remedy is easy: we just strip our concept of a rabbit (or electron) from all of these conceptual connections, which leaves us with a purely qualitative residue of rabbithood (or electronhood – though one might doubt whether anything at all is left over in this case). And then we can say that the mosaic contains rabbits (or electrons) in this watered-down sense. On reflection, however, this proposal really comes down to a *reduction* of rabbits and electrons to something ontologically more fundamental, much like the Lewisian claim that they reduce to material objects in his sense (i.e., to sums of point-sized bits of matter). In other words, we thereby leave substantial explosive realism behind, in favor of a more deflationary version of that theory.

So let us now consider that deflationary version of explosive realism. The mosaic, on this view, does not *fundamentally contain* all the different objects, as in substantial explosive realism, but rather *permits* being carved up into all those different kinds of objects (as Callender and Cohen indeed put it). One very straightforward way of giving meat to this idea is Lewis’s: his mosaic contains just material objects (in his sense), and these indeed permit to be ‘carved up’ in myriad different ways. One may define a kind of object *K* by just listing random qualitative and modal criteria; and if there are any material objects (in Lewis’s sense) fulfilling these criteria, then there are *K*’s. This works for rabbits (as Lewis thinks of them) just as well as it works for less familiar kinds of things. As long as a reductive story along the sketched lines can be told, anything can be claimed to be present in the mosaic, however modally ‘thick’ it may be. – But, now explosive realism seems to be nothing new: we are back where we started, with Lewisian metaphysics. The explosion thus doesn’t really add anything – it is indeed, in that respect, a rather deflationary sort of explosion.

Notice that both the substantial and the deflationary option seemingly provide the basics for the story about laws that Cohen and Callender are interested in: depending on our interests etc. we can approach nature (the mosaic) with a certain set of concepts (each science is warmly invited to bring its own set), and we will find that we can work with those concepts, do science with them (with more or less useful results). Armed with the concept *rabbit*, for instance, nature will show us all sorts of rabbit-related matters of fact. The difference between the two versions of explosive realism is that on the substantial view, objects corresponding to those concepts are among the fundamental ingredients of the mosaic, so that the rabbit-related matters of fact are irreducibly rabbit-involving, while on the deflationary view, such objects can (in principle) be reduced to the fundamental ingredients of the mosaic. And the latter is true also for Lewis’s own view. Deflationary explosive realism thus merely highlights a feature of the Lewisian view; it does not significantly depart from that view – at least, not insofar as the conception of the mosaic is concerned. (I consider whether this is enough for a ‘moderately’ Better Best Systems view in §3 below.)

Now, a genuinely new option is in the offing if we take the explosive realist idea in a radically different direction, i.e., if we decide to treat Lewis’s material objects, which form the reductive base that enables all the different carvings, as *itself* just the result of a carving operation. That is to say, we stop thinking in terms of a fundamental conception of the mosaic, providing a certain range of bottom-level, fundamental kinds of things (viz., Lewis’s point-sized bits of matter), to which everything reduces. By doing so, we suddenly find ourselves having left the camp of realism (at least, in one good sense of that slippery term): we stop asking for an account of the Humean mosaic, of what is ontologically fundamental, we simply say that every carving is the result of our applying concepts to *something* which also permits being carved up differently. – What is this ‘something’? To *what* exactly can we apply all those various sets of concepts? Well, there is no answer to this question, since every answer results from applying certain concepts. The view in question rejects the very idea of a fundamental conception of the mosaic: no carving is ontologically fundamental. And in that sense it is anti-realist. This view indeed really differs from Lewis’s account, which departs from the ontologically fundamental notion of point-sized bits of matter (in spatiotemporal arrangement).

It might be rather surprising that explosive realism, on this third reading, leads us straight to *anti*-realism. What is *called* ‘explosive realism’ turns out to be, at least on one reading of it, realism only by name.^26^ But what is more worrying, especially for proponents of a Humean theory of laws, is that the entire project now starts to break down. That is because the mosaic as it is independently of our carving it up has now become intractable – in contrast to Lewis’s view, on which there is a way of considering the mosaic independently from our way of carving it up, viz., in terms of the distribution of perfectly natural properties over the point-sized bits of matter. There is nothing to be said about the mosaic *independently* from our carving; the only way of saying something about it – for instance, that it exhibits certain regularities – is by *first* applying carving tools. So on this view it is, again, more appropriate to speak of *drawing* patterns into the mosaic than of *recognizing* patterns in it.^27^ Put differently: whereas in the previous options our choice between different vocabularies was a choice between focusing our attention on different aspects of the very same underlying reality, so to say, on this new, anti-realist view choosing between different vocabularies is more akin to choosing between different worlds – in Goodman’s words, it is choosing between different ‘ways of world-making’.

In order to see more clearly why this makes the Humean approach to laws of nature break down, we need to return to Lewis’s view on rabbits once more. We saw that he assumes that rabbits reduce to his material objects: a material object is a rabbit just if it displays a suitable arrangement of qualities *and* also has the right modal properties, which are spelled out in terms of its counterparts. Now, as I mentioned earlier, the counterpart relation itself depends on laws of nature, on Lewis’s view: the worlds in which our laws of nature are true are closer than the other worlds. They form the ‘inner sphere’. Hence the basis on which the laws of nature are determined cannot involve rabbits, or any other modally ‘thick’ objects – that would be circular; these modal features are to be understood in terms of the laws, after all. Again, the *fundamental* conception of the mosaic is in terms of the distribution of qualities over the modally ‘thin’ point-sized bits of matter. So Lewis’s conception of point-sized bits of matter, together with his array of purely categorical, perfectly natural properties, ensures that there is a decent basis for the Best Systems machinery to operate on. The freedom we may be said to enjoy as to how we want to ‘carve up’ nature can only be understood against the background of a fundamental conception of the mosaic that already provides the laws of nature. Such is the order of metaphysical dependence in Lewis’s system.

By contrast, our anti-realist reading of explosive realism leaves no room for such bootstrapping procedures. Given that no way of carving is ontologically fundamental, it is simply arbitrary to *first* carve into spatiotemporally individuated material objects (or point-sized bits of matter), *then* run a Best Systems machinery, and *then* do the rest of the carving. We could just as well simply carve directly into (modally thick) rabbits and the like, and then have the necessary connections we thus introduce play the role of laws of nature. The resulting view would be congenial to that more radical understanding of laws, which also derives from Hume: the projectivist approach that Nelson Goodman and others promoted.^28^ But of course, the point of the orthodox Humean, regularity-based account was that the regularities that are to be elevated to the status of laws are *objective*, mind-independent, and the resulting account of laws of nature therefore ‘admirably realist when compared against projectivism’ (Cohen and Callender 2009, p. 2).

We can now see that in order to arrive at a suitable objective and mind-independent (in short: realist) construal of the mosaic, one needs to endorse some kind of *positive* conception of the fundamental sort(s) of things that inhabit the mosaic. I have used a construal of the mosaic in terms of point-sized bits of matter displaying patterns of perfectly natural properties as my paradigm example, but strictly speaking this is not the only option. What matters is that the basic elements that occupy the mosaic (as well as the properties that are instantiated by those elements) are construed in a modally thin way – e.g., mereologically, spatiotemporally, or in some other way (if there is). We have seen that this rules out both substantial explosive realism and the anti-realist view just discussed. And I have indicated that the deflationary version of explosive realism does not really depart from a Lewis-style metaphysical view. However, as indicated, this may still seem to provide an entry point for the BBS theorist. In the next section, we consider this option in more detail.

## Moderately Better Best Systems?

Let us return to the question why the Humean should accept my assumption that rabbits, tables, water molecules, electrons etc. are modally ‘thick’ in the first place. Doesn’t this flagrantly beg the question against the Humean? Notice that I have valiantly admitted, and even stressed, that Humeans can deal with this alleged ‘thickness’ by offering a reductive account, for instance along the lines of Lewis’s story. (For the record, I am not in the least convinced that such a reduction can be pulled off – it will require, in Lewis’s version as well as in potential other versions, that we can fully separate off the modal from the qualitative, and I don’t think this is possible.^29^) But the crucial point here is that the very thought that rabbits, tables, etc. *can* be thought of in a modally thin way is nothing but a *statement* of Humeanism. To Humeans, this stripping off of the modal features of things is likely to be a matter of course (or a habit of the mind, if you like); if pressed, there will be allusions to arguments of a familiar Humean style (“you don’t *see* that this rabbit over here must have had parents, nourishment, etc., now do you?”). I will not discuss the credibility of these sorts of arguments here; it is enough for my purposes to point out that this skeptical attitude towards the modal features of things is not to be taken as the default understanding of objects in the world in the various sciences. It is by thus *not* following the Humean in reconceiving everything in modally ‘thin’ terms that I was able to point at possible conceptions of the mosaic that really do differ from Lewis’s. For my question was: what conception of the mosaic does this Humean stance require? It turned out that there is not much room indeed for diversity at this point: a Lewis-style fundamental conception of the mosaic, in terms of modally thin point-sized bits of matter, or something along those lines, is going to be the standard for the Humean. So no adventurous explosion of reality will be available, and the generous gesture towards special sciences, to simply bring their own conceptual toolbox to the game, turned out to come under special conditions: the conception of the *objects* that form the domain to which the predicates of the relevant science are applicable must be ‘Humeanized’.

Now, the reader might at this point be left with a seemingly justified puzzlement: if what I’ve been putting forward makes sense, there is no room for a substantial departure from the Lewisian orthodoxy, but I also hinted that this orthodoxy comes close to being simply a ‘statement of Humeanism’.^30^ So isn’t it enough, then, for a BBS theory to simply stick to the Lewisian story, and merely insist that we give up the doctrine of perfectly natural properties when it comes to running a Best Systems competition? I even admitted that one could still justifiably characterize the change in view thus proposed as a move towards ‘explosive realism’ – if one thereby means the deflationary version of that doctrine explored above.

This points in the direction of the Humean option that my considerations in §2 left open. It can be made precise as follows.^31^ Suppose that we do start with a set of sortal concepts that may come with ‘thick’ modal profiles – for instance, suppose we start with rabbits and the like. (Perhaps we are zoologists.) Now, I said, this threatens to make the detour through a Best Systems machinery superfluous: we already have all manner of ‘necessary connections’ built into those modally ‘thick’ concepts. But, so the Humean might argue, we can understand these to be provisional. We can simply use those concepts with all their modal thickness to merely *fix their extensions*, and then run a Best Systems competition on the basis of those extensions. The laws resulting from this procedure might exactly mirror the modal connections we started out with, but they need not. Perhaps they mirror them only partly, perhaps no laws are forthcoming whatsoever, and perhaps unexpected new ones emerge.

Notice that in order to “fix extensions”, we need to have a conception of the entities that will make up those extensions – no entity without identity, as Quine famously quipped. As the point of the extension fixing is to get rid of the modal ‘thickness’, the conception of the elements in those extensions must be like Lewis’s: modally thin. In other words, we are indeed back with deflationary explosive realism.

The proposal is, thus, to first ‘convert’ the set of concepts of a given science (zoology, in our example) to Humeanly acceptable ones, and then run a Best Systems competition on that basis. This conversion comes down to a reductive account of the relevant modally thick kinds. Thus, rabbits are supposed to be reducible to material objects in a suitably modally thin sense (e.g., sums of point-sized bits of matter) displaying appropriate patterns of qualities, and standing in suitable modal relations.

Now, as I have stressed above, this assumed reduction is not a matter of course. In particular, such a reduction is likely to require perfectly natural properties. For without those, the reductive claim has little substance. If all sets of point-sized bits of matter (or all sets of material objects, for that matter) are equal as regards their ‘naturalness’, there is no such thing as *the* pattern of qualities corresponding to any particular rabbit, much less anything like a pattern of qualities that is (somehow!) common to all rabbits. So we would first need to isolate some set of relevant properties in terms of which the reduction may proceed. And this set would in fact be available if the science in question (zoology, we’re imagining) came with its own privileged set of fundamental properties in terms of which the reduction is to be effected. But, again, this is simply incredible: no special science comes pre-packaged with its own Humeanly acceptable reductive apparatus.

Now, the Humean might simply hold that the qualitative aspect of, e.g., rabbits requires no such reduction to patterns of more basic qualities, that we can treat *rabbit*, when stripped of all modal elements, as simply an irreducible feature of certain material objects considered as wholes, i.e., as a ‘perfectly natural property’ in its own right.^32^ Now, further doubts could be articulated about the feasibility of this proposal. For instance, this is not likely to be faithful to the way *rabbit* is treated in zoology. And we are still assuming that rabbits can be reductively conceived in such a way that their modal dimension is disjoint from their qualitative dimension, yet this assumed disjoint conception is likely to presuppose laws – laws are to determine which possible worlds are relevant for the evaluation of the modal dimension, after all. Perhaps this can in turn be solved by insisting that the reduction doesn’t require a full-blown *analysis* of the modality involved, but merely a *removal* of that modality. Well, maybe; but notice that this again adds to the list of demands the defender of a BBS approach is putting on the sciences to be allowed into her game in the first place. Only if one assumes *both* a modally flat conception of the underlying mosaic, *and* a reductive analysis of the science’s basic vocabulary along Humean lines which does *not* already invoke laws of any kind, is there any chance of getting the proposal off the ground.

I will not pursue this further as an argument against the BBS approach, but leave it to the reader to judge whether there is reason for optimism. It was not my aim, after all, to refute this view, but rather to explore what the conception of the underlying mosaic must be like in order to be acceptable for such a Humean project. So let us zoom out a bit and see what kind of answer our reflections suggest to that larger question.

## Afterthoughts: the limits of Humeanism

Our explorations give rise to the following diagnosis. The misgivings about ‘necessary connections’ that are more or less definitive of Humeanism are, in the end, symptoms of a very deeply rooted suspicion towards *conceptual* connections in general: conceptual connections are, at best, mere relations between ideas, to put it in Humean terminology; what could they possibly tell us about reality? To say that they capture real ‘necessary connections’ out there is just to project what belongs within the mind to the outside world. However, and this is what I have tried to bring out in the above, to cash out this familiar Humean picture, the Humean needs to devise a conception of this ‘outside world’ that indeed lacks those necessary connections. Put briefly, the Humean strategy of ‘disarming’ conceptual connections by showing that they amount to no more than harmless, matter-of-factly regularities among the things ‘out there’ requires that the fundamental conception of those things ‘out there’ be devoid of conceptual connections. So the Humean project requires that the fundamental conception of the mosaic be modally ‘thin’, in order to provide the basis in terms of which the more troublesome conceptual connections can be disarmed. I took Lewis’s view as my stock example in the above, and argued that something along those lines is what is required.

This diagnosis thus indicates a ‘limit of Humeanism’: the options for a credible conception of the Humean mosaic are quite limited – e.g., a shift to an ‘explosive realism’ can only be made within these limits (i.e., in the form of ‘deflationary’ explosive realism). But we can push this conclusion a little bit further, albeit tentatively, to indicate a more troublesome ‘limit of Humeanism’: if it is reification of conceptual connections that is bothering the Humean, there is considerable pressure for him to resort to anti-realism. For even on as modally flat a conception of the Humean mosaic as Lewis presents, there are still conceptual connections: his spatiotemporally (or mereologically) individuated material objects come with their own humble but non-empty modal profiles. For instance, no material object could be larger or smaller than it is, since different spatiotemporal boundaries *by definition* means different material object.^33^

Let me close by linking these observations to the traditional Humean argument against necessary connections. Hume says that, if we look closely, we never *see* any necessary connections – this is an expression of the mentioned suspicion towards conceptual connections.^34^ By extension, however, we could argue that we never *see* any succession in time or any contiguity in space; we only see the sensory qualities, the spatial and temporal relations between them are not *themselves* such qualities. Indeed, even the *difference* and the *resemblance* between different sensory impressions are not themselves impressions, nor is the conceptual determination of impressions *as impressions* an impression.^35^ Thinking this traditional Humean argument through to the end, then, leads to the conclusion that all of these determinations are the result of what the mind does with ‘the given’, much like the mind, on Hume’s view, creates an expectation of the effect whenever the cause is present. At its limit, then, this type of suspicion towards the conceptual leads to a form of anti-realism, on which *no* fundamental conception of the mosaic can be given at all. At this limit, Humeanism entails rejection of metaphysics *tout court*, and hence will not yield an ‘admirably realist’ account of anything.

## Footnotes

1 An anonymous referee reminded me that Roberts (2008) claims to be able to marry Humeanism to ‘governing’ (see, esp., §10.5). His is, however, a rather peculiar type of governing – and I would submit that it is questionable whether his basic notion of ‘legitimate measurement procedures’ is in the end consistent with Humeanism. Showing this would of course require a detailed discussion of Roberts’s views, which I cannot undertake in the context of this paper. So when I talk of Humeans and Humeanism, I intend to be talking about mainstream or standard Humeans and Humeanism, and leave it open whether my considerations are relevant for less ‘orthodox’ variants like Roberts’s.

2 This epistemological take on the Humean mosaic later became the guiding thought of phenomenalism and of logical empiricism. Hume himself can be taken to have started that tradition – see esp. Hume (2010, Part IV); but probably Mill (1865, chs. XI–XIII) contains the first thorough statement of phenomenalism.

3 Of course, this does not mean that contemporary Humeans have nothing to say on, e.g., mental causation. That is to say, they will claim that among the mind-independent matters of fact there are those that are (or constitute, or subvene) mental states and events.

4 I follow Lewis’s phrasing here in speaking of the “Humean mosaic”. Other labels can also be found in the literature; Roberts, for instance, calls it the “Humean base” (Roberts 2008, p. 353).

5 We should not misunderstand Lewis here: he is not defending the physics that seems to be presupposed by his present formulation. In a later article he makes explicit that he defends ‘the *philosophical* tenability of Humean Supervenience,’ and that his defense ‘can doubtless be adapted to whatever better supervenience thesis may emerge from better physics’ (Lewis 1994, p. 226).

6 For instance, Braithwaite (1927, 1928) suggested to understand the laws to be those generalizations that are ‘believed for reasons that are not logically demonstrative’ (1927, p. 467), that is, not deductively derived from particular matters of fact. A contemporary heir to this sort of suggestion is Swartz, who summarizes his position as follows: ‘there are a number (an infinite number, perhaps) of true universal, material conditional statements (propositions), all of whose terms are perfectly descriptive, that is, make no reference to any particular time, place, person, or thing in the world. The Regularist is content to allow that all these true universal, material conditionals are physical laws …’ (Swartz 1985, p. 101). For an excellent in-depth discussion of the development of Humeanism over the past century, and the challenges it faces, see Stroud (2011, Ch. 2).

7 Apart from Lewis himself, defenders of the Best Systems approach, in one or another version, include Beebee (2000), Albert (2001), Earman and Roberts (2005a, b), Loewer (2012), Dunn (2011), Cohen and Callender (2009), and Callender and Cohen (2010).

8 Of course, it will be crucial to the case for a Best Systems approach that principled accounts of simplicity and strength, and their balance, can be given. However, for present purposes we can abstract away from those details and the worries they may evoke. (Such as the well-known worry that these norms incorporate an unwanted psychological aspect into what it is to be a law. Lewis thinks not, as long as ‘nature is kind enough’ to convincingly make one system the absolute best one – see Lewis (1994, p. 479).)

9 Lewis does not think that ‘there are laws that essentially mention Smith’s garden, the center of the earth or of the universe, or even the Big Bang’, but still, ‘such laws ought not to be excluded a priori’ (Lewis 1986b, p. 123). Albert (2001) and Loewer (1996) make use of this option in arguing that we should include the ‘Past Hypothesis’ (viz., that the universe started out with a very low entropy state) in the Best System. See Frisch (2011) for critical discussion.

10 See also Lewis (1983, pp. 366–8) and Lewis (1986b, pp. 123–4). Lewis envisages three options for privileging natural properties: by means of a primitive predicate of naturalness; by providing a reductive analysis in terms of universals; or in terms of tropes. But he does not take a stand: ‘the honors are roughly even, and remain undecided’ (Lewis 1986a, p. 64).

11 But see Taylor (1993) for such a theory-relative (and hence anti-realist) construal of naturalness.

12 This point is developed in detail by Cohen and Callender (2009, pp. 5–8).

13 For a clear statement of just how Lewis intends this identification to be read, see Lewis (1994, esp. p. 474).

14 See Schrenk (2006) and Cohen and Callender (2009), Callender and Cohen (2010). The label ‘Better Best Systems’ is Callender and Cohen’s.

15 Interesting questions can be asked about the proposal. For example, in biology most of the vocabulary from physics is also used, but it seems possible that some law of physics, resulting from a physical vocabulary based Best Systems competition, will not be a law in the Best System to which biology’s vocabulary gives rise (or vice versa). I will not discuss these issues here, however.

16 Some Lewisians take this to be the natural option, for reasons of parsimony: “The identification of spatiotemporal objects with the regions is just crying out to be made” as Sider (2001, p. 110) writes, because “otherwise we seem to gratuitously add a category of objects to our ontology.”

17 Lewis adopts classical extensional mereology – see, e.g., Lewis (1991, p. 81): “Given a prior commitment to cats, say, a commitment to cat-fusions [mereological sums of cats; JM] is not a *further* commitment. The fusion is nothing over and above the cats that compose it. It just *is* them. They just *are* it. Take them together or take them separately, the cats are the same portion of Reality either way.”

18 There is an apparent conflict here between the modal features to which Lewis’s principle of individuation for objects gives rise (different region/parts, different object) and his counterpart-theoretic account of modality. See Mulder (2013) for discussion.

19 This assumption mirrors, of course, Hume’s famous distinction between matters of fact and relations of ideas.

20 In Mulder (2013), I develop an account of the notion of fundamentality I am using here, and of the related notion of reduction. (Following an anonymous referee’s advice, I have abstained from using the term “supervenience” here: it invites difficult questions that I don’t want to go into. Still, I think that what Lewis aimed to capture with his label “Humean Supervenience” comes very close to what I here sketch in terms of reduction and fundamentality.)

21 Cohen and Callender borrow explosive realism from Sosa (1993, 1999), Dorr (2005), and Eklund (2008), amongst others.

22 Stroud diagnoses this ‘blind spot’ in what he calls the ‘more or less standard picture’ underlying much contemporary Humean thought as a result of adopting ‘first-order quantification theory’: this logical theory makes it seem as if we can have a domain of objects without having to bother about how we conceive of those objects – see, esp., Stroud (2011, p. 27).

23 An anonymous referee made it clear to me that a Humean is likely to reject this rendering of the position out of hand by stating that it assumes without argument that things like rabbits, tables, water molecules etc. really *are* modally ‘thick’, and that it would be much more plausible to start with a less ‘inflated’ conception of those things. I briefly consider this option further down, and return to it once more in §3.

24 Here my notion of fundamentality is crucial – again, see Mulder (2013) for more detailed discussion.

25 We may of course go on to systematize these connections in a way much like the Best Systems approach proposes, but it is clear that this does not even come close to being a Humean approach to laws of nature.

26 I have been sloppy here with regard to how we should understand “realism”. I do not think my argument requires that I be more specific, but it may be helpful to point out that in the present context, there is at least a clear distinction to be drawn between a regularity-based account of laws that takes the regularities to be “there anyway”, whether or not we single them out, and a regularity-based account that takes the regularities to depend on us. The former is an ingredient in Lewis’s realism; the ‘new option’ I am considering here rejects this and insofar can be considered an ‘anti-realism’.

27 These considerations have taken us in the direction of a ‘conventionalist’ view on material objects like Sidelle’s, who calls what exists independently of our carving “pure, non-individuated stuff”. See Sidelle (1998, 2010). I seriously doubt whether this view is coherent, but that is beside the current point.

28 See, e.g., Goodman (1955) and Ward (2002).

29 See, e.g., Millgram (2009, 2011) for powerful suspicions about this sort of reduction generally; and Mulder (2016) for a defense of the irreducibility of the basic categories of biology in particular. – Also, as I noted earlier, the presupposed separability of the qualitative and modal dimension of things resembles Hume’s famous division between matters of fact and relations of ideas.

30 Thanks to an anonymous referee for insisting that I treat this option in more detail.

31 I owe awareness of this interesting further Humean move to an anonymous referee, who pointed out to me that Markus Schrenk, one of the earliest defenders of a Better Best Systems view (see Schrenk 2006), discussed it during a talk entitled “Trouble with Properties for Better Best Systems” at LMU on 17 December 2012.

32 An anonymous referee suggested that I discuss the view of rabbithood as a quiddity at this point. I won’t do so, since I do not want to get involved in the metaphysical intricacies that this notion invites.

33 See Mulder (2013) for a more elaborate defense of this claim, and for treatment of the obvious reply that Lewis can solve this with the aid of his counterpart-theoretic analysis of modality.

34 Stroud (2011, p. 95) rightly remarks that this famous Humean line of thought is indeed not really an argument for, but rather an expression of, his skepticism. See also Anscombe (1971).

35 These remarks aim to bring out that also in the context of this traditional Humean thought – “you don’t *see* necessary connections” – an implicit commitment to some sort of *conception* of what it is that we *do* “see” can be discerned. And this conception itself of course incorporates conceptual connections.
